# Secondary Microsurgical Reconstruction of the Cervical Esophagus: Safer Flaps and Practical Tips in a Challenging Situation

**DOI:** 10.3390/jcm13092726

**Published:** 2024-05-06

**Authors:** Vittorio Ramella, Andrea Ferrari, Federico Cesare Novati, Zoran Marij Arnež, Grace Marchi, Agostino Rodda, Stefano Bottosso, Giovanni Papa

**Affiliations:** 1 Plastic Surgery Department, Ospedale di Cattinara, ASUGI (Azienda Sanitaria Universitaria Giuliano Isontina), 34149 Trieste, Italy; 2 Department of Medical, Surgical and Health Sciences, Plastic and Reconstructive Surgery Unit, University of Trieste, 34149 Trieste, Italy

**Keywords:** esophagus, cervical, secondary, reconstruction, microsurgery, flap, ALT, radial, parascapular

## Abstract

**Background**: Cervical esophageal reconstruction is vital to improve the quality of life in cancer surgery patients. Microsurgery is crucial in providing vascularized tissue for defect repair, particularly in secondary cases with a higher risk of failure due to larger defects and damage from previous surgery and radiotherapy. The purpose of this study was to describe the clinical characteristics of a series of patients who underwent secondary repair of esophageal defects and provide practical information for the management and treatment of such cases based on the authors’ experience and the literature review. **Methods**: We retrospectively reviewed the electronic medical records of the Plastic Surgery Clinic at the University of Trieste to identify cases of patients who underwent secondary esophageal microsurgical reconstructions following oncological surgery. Patient demographics, the etiology of esophageal defects, previous surgical history, and preoperative assessments were collected from medical records. Surgical techniques utilized for reconstruction, such as pedicled flaps or free tissue transfers, were documented along with intraoperative information. Postoperative outcomes, including complications, graft viability, and functional outcomes, were evaluated during follow-up. **Results**: We treated 13 cases of secondary esophageal reconstructions between 2011 and 2022. Most commonly, Antero-Lateral Thigh (ALT) flaps were used in 10 cases, while 2 cases employed a radial forearm flap (RFF), and 1 case employed a chimeric parascapular flap. No flap failures occurred during a median 50-month follow-up. One ALT flap patient experienced postop stricture but maintained swallowing ability. A single tracheoesophageal fistula occurred in an RFF patient with a history of radiotherapy and complete lymph node dissection. **Conclusions**: Cervical esophageal reconstruction significantly impacts patients’ quality of life by restoring oral feeding and phonation. When local flaps fall short, microsurgical reconstruction with intestinal flaps is valuable but is burdened by limitations. For challenging secondary cases, ALT or RFF flaps emerge as safer options due to their robust pedicles, yielding low complication rates and positive functional outcomes.

## 1. Introduction

Several standard treatment choices following upper gastrointestinal tract interruption, like gastrostomy and jejunostomy, have been documented in the literature [1]. However, they determine adverse sequelae related to the elimination of saliva, malnutrition, aspiration pneumonia, and erosion of the skin around the stoma. This significantly impacts the patient’s quality of life and ability to work. Individuals lacking esophageal reconstruction are unable to consume food orally or swallow saliva, necessitating a constant expulsion of approximately 0.6 L of saliva daily, even during the night-time [2].

Reconstruction of the esophagus and restoration of the GI tract is therefore mandatory to re-establish an acceptable quality of life. Conventional surgical techniques such as gastric pull up or locoregional flaps could represent valid alternatives in primary cases [3]. However, their feasibility depends on tissue damage and defect size, and they are contraindicated in secondary reconstructions.

Microsurgical reconstructive procedures represent the most effective solution to date. Among these, intestinal flaps including the jejunum and colon free flaps [4,5] have historically been considered the standard for esophageal reconstruction [6]; in addition, microsurgical flaps may be employed for additional food intake pathways’ creation [7]. Jejunum flaps have shown a low stricture rate in circumferential pharyngeal defects compared with other microsurgical free flaps [8], and intestinal flaps can be successfully used in secondary cases as well, after the failure of primary reconstructions, in order to guarantee esophageal continuity [9].

However, there are some divergent indications and complications associated with intestinal flaps the surgeon sometimes has to face to avoid complications associated with intestinal flaps, such as restricted flap/pedicle length in relation to reconstructive necessities, abdominal complications, prolonged swallowing (resulting from delayed bolus transit, dysmotility, and asynchronous peristalsis), as well as the potential for vascular disruption due to arteriosclerotic modifications and brief ischemic periods.

In light of this, fascio-cutaneous flaps can represent a more accessible option, sometimes being even safer, in selected cases of both primary and secondary reconstruction [10].

**Figure 1:**
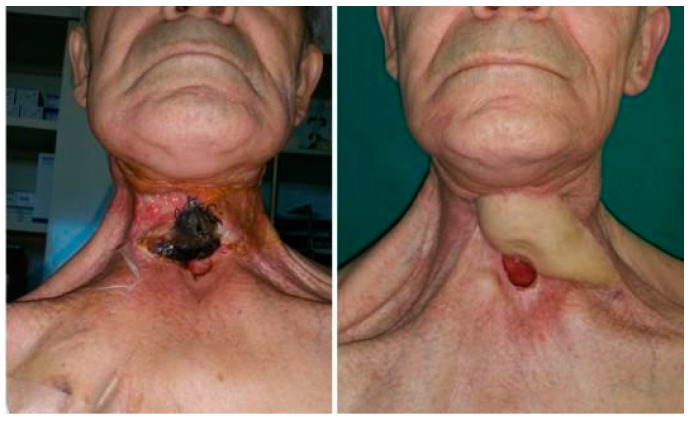
Secondary reconstruction with ALT flap in patient with large skin defect.

**Figure 2:**
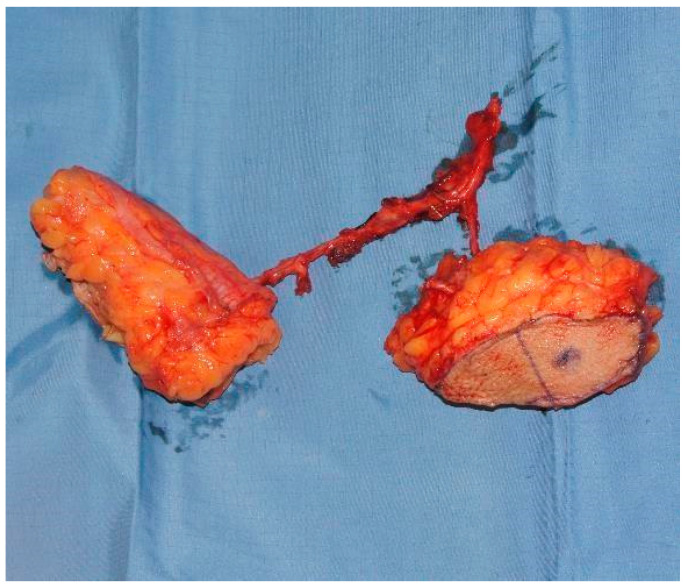
Double-island flap for esophageal reconstruction with skin paddle based on further perforators.

Concerning the donor site, fascio-cutaneous flaps may offer a more effective solution. Through a collaborative double-team approach, fascio-cutaneous flaps can be harvested simultaneously with the preparation of the recipient vessels. This allows them to be easily shaped to precisely fit the soft tissue defect that requires coverage. Additionally, secondary cases, often resulting from the failure of previous surgeries and typically associated with esophago-tracheal fistulas or complicated tracheostoma, present challenges related to skin coverage of the anterior cervical region (Figure 1). In this context, fascio-cutaneous flaps also allow the design of a double-island flap based on several perforators, one for the reconstruction of the esophageal tract and the other for the cutaneous coverage of the neck (Figure 2). Finally, this procedure also allows the evaluation of the viability of tubularized submerged flaps [11].

To date, the literature lacks recent studies that thoroughly examine the clinical attributes of different treatment options, along with practical insights to aid healthcare practitioners in their decision-making process.

The aim of this study was to describe a cohort of patients who underwent the secondary repair of esophageal defects due to the failure of immediate reconstruction following the ablation of cervical esophageal cancer, while also providing pragmatic insights for the effective handling and therapeutic approaches in such scenarios, through both the authors’ expertise and a comprehensive literature analysis.

## 2. Patients and Methods

We retrospectively reviewed the electronic medical records of the Plastic Surgery Clinic at the University of Trieste to identify cases of patients who underwent secondary esophageal microsurgical reconstructions following oncological surgery between January 2011 and September 2022. The study protocol was approved by the University of Trieste Ethics Committee on Clinical Investigation in compliance with the Helsinki Declaration. All patients signed an informed consent form.

The study included all consecutive patients that met the following inclusion criteria: (a) age ≥ 18 years; (b) previous surgery for cervical esophageal carcinoma; (c) secondary esophageal microsurgical reconstructions; (d) minimum follow-up of 9 months.

Based on our institutional policy, the following parameters were considered guidelines for the reconstructive choice: (a) general status and comorbidities; (b) previous or programmed radiotherapy of the cervical region or neck; (c) CT angiography evaluation of defect dimensions, available space at the cervical level, and quality of remaining neck vessels after oncologic surgery; (d) flap availability; (e) nutritional status; (f) skin pliability for fascio-cutaneous flaps. Following this evaluation, our options usually included three types of flaps: the Antero-Lateral Thigh (ALT) flap, the radial forearm flap (RFF), and the parascapular flap. The first two represent the primary choices in our institution, while the parascapular flap is selected as a backup option in case the first two are not feasible.

**Figure 3:**
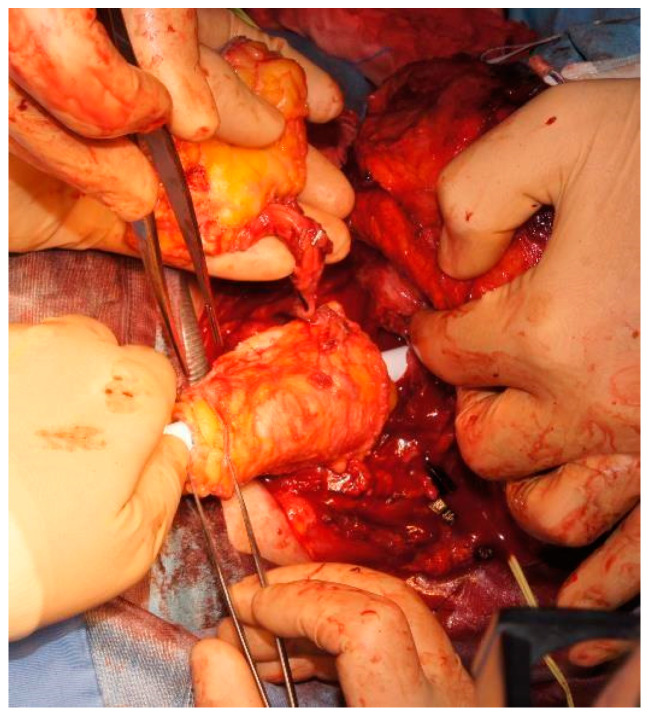
Flap tubularized on a self-expandable plastic stent.

At our institution, all patients eligible for secondary esophageal reconstruction with microvascular flaps receive prophylactic antibiotic, antithrombotic, and anti-reflux treatment during the perioperative period. Furthermore, they are planned to have a nasogastric tube and a self-expandable plastic stent (SEPS) positioned within the tubularized flap to allow placement at the cervical level, in order to prevent wrinkling and potential kinking (Figure 3). The device is kept in place for the first 5 days at least. In our experience, younger patients with trophic tissues and higher salivation could benefit from keeping the SEPS in place for a longer time (over 7 days), due to the higher risk of salivary leaking and subsequent dehiscence or fistulization. All flaps are monitored every hour in the first 48 h after surgery, and then every 3 h until the end of the first week. In all flaps with an external skin island, the following parameters are evaluated: color, temperature, capillary refill, texture, and arterial flow assessment via Doppler probe. Fully buried flaps are monitored via the Licox^®^ (Integra LifeSciences; Princeton, NJ, USA) PtO2 mini-invasive monitoring system or via a Synovis Flow Coupler^®^ (Synovis Micro Companies Alliance; Birmingham, AL, USA) Doppler device placed on the venous anastomosis when available.

**Figure 4:**
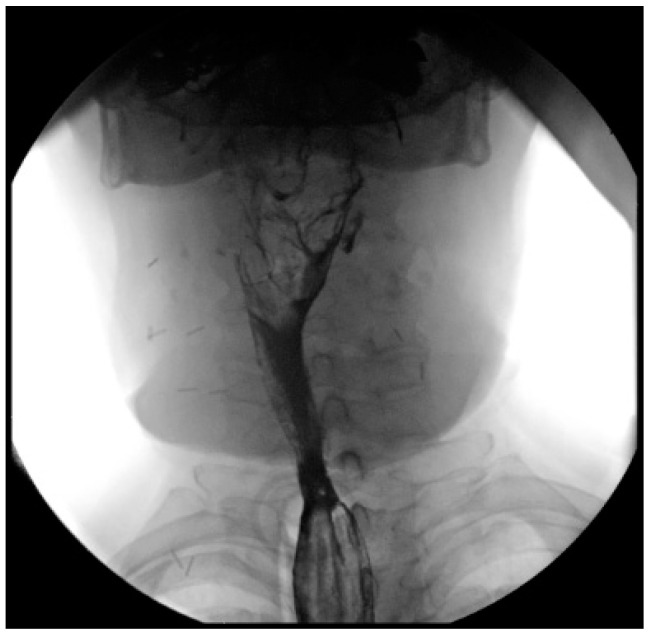
Barium meal after ALT flap reconstruction to assess transit function.

Patients are assessed for the presence of fistulas, esophageal strictures, and transit function via a barium meal 30 days after surgery (Figure 4).

## 3. Results

From 1 January 2011 to 20 September 2022, 13 patients underwent secondary esophageal reconstructions due to the failure of immediate reconstruction following the ablation of cervical esophageal cancer: median (IQR) age at surgery, 58 (51–71) years; 10 (76.9%) males (Table 1). The median hospitalization time was 16 days.

Seven patients exhibited pharyngo-cutaneous fistulas, five experienced dysphagia due to pharyngoesophageal strictures, and one patient had a history of disease recurrence after cordectomy and subsequent total laringectomy with first reconstruction failure, which occurred prior to coming to our attention. All 13 patients had a history of prior radiotherapy, and 7 had undergone complete lymph node dissection. Eleven patients had a history of alcohol consumption and nine were former smokers.

We chose an ALT flap in 10 cases, 6 of which was with a dual paddle, 1 was tubularized for GI tract reconstruction, and the other for neck skin coverage. RFF was used in two cases, while one patient was treated with a chimeric parascapular and scapular flap with a double skin paddle. All ALT flap donor sites and the parascapular flap donor site were repaired via primary closure, while RFF donor sites were covered via a split-thickness skin graft.

During a median follow-up of 50 months, no flap failures were observed. One patient developed postoperative stricture after ALT flap reconstruction; however, he still maintained the ability to swallow semi-liquid foods. In this patient, hypopharyngoscopy revealed a distal displacement of the SEPS towards the stomach. The patient required further mechanical dilation procedures after discharge to improve the functional outcome. This observation highlights the importance of positioning an appropriately sized SEPS to reduce the risk of subsequent descent and postoperative stricture.

Additionally, there was a singular occurrence of tracheoesophageal fistula, attributed to the tracheal tube, in a former smoker who underwent RFF reconstruction and had a history of previous radiotherapy and complete lymph node dissection.

## 4. Discussion

Secondary cases of cervical esophageal reconstruction can be challenging due to the extensive damage to soft tissues, large defect size after debridement, and the poor conditions of recipient vessels due to previous surgery and radiotherapy [12]. In this context, locoregional flaps are usually not a suitable option, while intestinal flaps come with a high risk of complications in patients whose general status is already compromised [13] and may not be able to guarantee full-length coverage and/or a needed skin paddle as well. Therefore, these patients require a thoughtful approach, with safe techniques and the performance of extensive preoperative examination being preferred [14]. The use of intestinal flaps such as the jejunal free flap can lower stricture rates, and it has shown reliability in secondary cases as well. However, in our experience, fascio-cutaneous flaps may ensure greater reliability in terms of coverage capacity, tissue resistance, and the management of distance from recipient vessels [8]. CT-angiography guides the choice of the recipient vessels for anastomosis, especially in patients with previous cervical lymph node dissection and/or radiation. Recipient vessels in esophageal reconstruction with free flaps usually include the facial artery or the superior thyroid artery, but in secondary cases, these vessels could be damaged or unsuitable. It is a great advantage to have a long pedicle length available, allowing for distant anastomosis, even on extra-regional vessels such as the thyrocervical trunk, the transverse cervical artery or the thoracoacromial artery, which represent a good choice in the case of post-radiation cervical damage.

The evaluation of the affected area is necessary to design a tissue flap that can accurately match the volume of the defect: given the anatomical complexity of the region and when aiming for a functional reconstructed tract, there should not be any excess tissue both in the neck skin externally and internally at the level of the two circular sutures positioned distally at the esophageal stricture and proximally at the tongue base. Excess tissue near the anastomosis between the flap and the distal portion of the esophagus can ripple, creating saliva stagnation and subsequent fistulas and suture dehiscence. Moreover, the reconstructed cervical esophagus is prone to excoriation by the tracheal cannula curvature, especially when rigid cannulas are used, therefore promoting fistulization [15].

The most suitable flap must then be chosen according to its thickness, its structure, and the available space at the cervical level, as well as donor-site morbidity. In patients with adequate skin coverage of the anterior neck, we evaluated all those characteristics to guide our decision. In one case, we began to harvest an ALT flap but intraoperative assessment during dissection did not show adequate perforators; thus, we harvested an RFF. In all patients presenting skin defects of the cervical area, the RFF was deemed unsuitable for the reconstruction.

Generally, our first choice for secondary esophageal reconstruction is the ALT flap, followed by the RFF and the parascapular flap.

### 4.1. Anterolateral Thigh Flap

The ALT flap is widely used in the reconstruction of head and neck defects [16], and it is our first choice when the expected thickness of the flap is less than 2 cm. ALT flaps allow a long pedicle to reach the recipient vessels even in unconventional sites, and they can be designed as chimeric flaps, including a portion of vastus lateralis muscle for the protection of the great vessels or with a second skin island for skin coverage of the neck. They also allow the harvesting of a large portion of fascia which provides greater protection of the sutures and leakage prevention [17].

An ALT flap can be harvested simultaneously while a second team is working on the cervical district. Its pedicle enters laterally once the flap is set in the cervical region and it can be easily positioned where needed to reach distant recipient vessels. In the case of tubularized ALT, the flap insetting and suturing of the posterior area can be challenging because of the firm texture of this flap; hence, sometimes, it can be indicated to suture the proximal and distal circumferences first, and then to tubularize the flap and to suture its long axis. We used this modified technique in four cases. Some other authors prefer to suture the proximal/pharingeal aspect first, then to tubularize the flap, and finally to secure the distal/thoracic circumference [18].

### 4.2. Radial Forearm Flap

The radial forearm flap (RFF) is our second choice for secondary esophageal reconstruction. Therefore, it was used when the ALT flap turned out to be too thick, making it unsuitable for an optimal tubulization, or its perforators were deemed insufficient. However, because of its thinner dermis, the RFF does not provide the same sturdy shape once tubularized as the ALT flap, which is why greater precision must be applied when designing and harvesting the flap. In fact, it is mandatory to obtain exact shapes and dimensions, not exceeding the tissue defect length, to avoid kneeling or the creation of pouches that can collect saliva and then fistulize. For this reason, fistulization rates are higher in RFFs than in ALT flaps in the literature (RFF 17–67% vs. ALT 0–13%) [19], as well as stricture rates [20]. The pedicle of the RFF is very long and sturdy, with two available venous drainage systems. However, because the pedicle enters the flap axially, it can only be positioned superiorly or inferiorly when the flap is placed to cover the defect, and this requires thoughtful programming, as it may not fall in the preferred location compared to the ALT perforator flap. This characteristic is particularly limiting in secondary cases, due to the usually scarce or damaged recipient vessels.

### 4.3. Parascapular Flap

The parascapular flap is a safe flap, and it can be designed with a double skin paddle [21,22], or even as a chimeric flap with a portion of latissimus dorsi muscle [23]. However, its application is limited because its dermis is usually very thick and rigid and therefore difficult to be tubularized in place, making this flap inadequate in the case of insufficient room in the recipient site. The surgery is also complicated by patient positioning, requiring the patient’s rotation on the table to harvest and then inset the flap.

For this reason, we kept it as a salvage flap in one single patient when the other flaps were unavailable as they had already been used for other reconstructions with subsequent failure. In this case, we performed a double-island scapular and parascapular flap, as we needed two adequate skin pads, the first to reconstruct the cervical esophagus and the second to repair a skin defect subsequent to complicated tracheostoma.

### 4.4. Surgical Tips

All these flaps should be tubularized with the skin facing the internal lumen. For this reason, the flap must be designed slightly larger than required, since the “reverse” rotation is somewhat spoiled by the dermis, resulting in a more difficult rotation in this direction.

It must be noted how the shape of the cervical esophagus design is trapezoidal instead of cylindrical: the mean diameter at the tongue base is usually about 4–5 cm, while it is smaller inferiorly, about 3 cm, with a structure colliding on itself in a virtual esophageal lumen.

In our experience, we prefer not to place the longitudinal suture of the flap posteriorly, as the posterior area is most prone to collections and possible fistulization; however, some authors have different opinions on the matter, and prefer to place the vertical suture posteriorly, facing the prevertebral fascia [24].

Multiple layers of suture should be used, at least three: a cutaneous suture positioned on the lumen side, a subcutaneous suture on the dermis, and then one on the fascia that should be precisely harvested with a larger area than the cutaneous island, to create an outer layer of tissue protecting the first two.

During tubularization, the vertical suture line can be overlapped with de-epithelialized flap skin edges along with invagination of the esophagostoma into the skin tube to cover the end-to-end anastomosis with soft tissue.

A vertical incision of the proximal esophagus allows the ‘key’ of the skin flap to expand the caliber of the anastomosis and break up the circumferential suture line in an attempt to prevent contraction and stenosis [25].

The pedicle entry site must also be carefully chosen so that it is placed on one of the two sides of the flap, avoiding the posterior and superior areas that are prone to crushing; the side of choice must then coincide with the side of the receiving vessels.

## 5. Conclusions

The secondary repair of esophageal defects after the failure of immediate reconstruction following the ablation of cervical esophageal cancer represents a reconstructive challenge due to extended tissue damage and poor recipient vessels. In this context, safer flaps with long and sturdy pedicles represent a good choice in our experience, with the ability to provide a skin paddle; low complication rates in terms of flap failure, fistulas, and neo-esophagus stricture; and good functional outcomes as well.

## Figures and Tables

**Table 1:** Demographics and complications.

	Age	Sex	Flap Used	Donor Site	Post-Op Fistula	Post-Op Stricture	Follow-Up (Months)	Hospitalization Time (Days)
1	57	M	ALT	Primary closure	no	no	146	14
2	58	M	ALT w/ double skin paddle	Primary closure	no	no	120	20
3	64	M	ALT	Primary closure	no	no	111	17
4	51	M	ALT w/ double skin paddle	Primary closure	no	no	86	14
5	57	F	RFF	Skin graft	no	no	72	19
6	61	M	ALT w/ double skin paddle	Primary closure	no	no	56	12
7	54	M	ALT w/ double skin paddle	Primary closure	no	no	50	15
8	71	M	ALT	Primary closure	no	Mild (oral feeding possible w/ semiliquid diet)	46	31
9	56	M	ALT	Primary closure	no	no	39	12
10	62	F	Scapular w/ double skin paddle	Primary closure	no	no	30	19
11	52	M	ALT w/ double skin paddle	Primary closure	no	no	21	16
12	66	M	RFF	Skin graft	Yes	no	17	38
13	63	M	ALT w/ double skin paddle	Primary closure	no	no	12	12

## Data Availability

The data presented in this study are available on request from the corresponding author due to privacy issues.
